# The Effects of Smoking on Expression of IL-12 and IL-1β in Gingival Tissues of Patients with Chronic Periodontitis

**DOI:** 10.2174/1874210601711010595

**Published:** 2017-11-24

**Authors:** Amir Moeintaghavi, Hamid Reza Arab, Seyed Abdol Rahim Rezaee, Hani Naderi, Farid Shiezadeh, Saber Sadeghi, Najme Anvari

**Affiliations:** 1 Dental Research Center, Mashhad University of Medical Sciences, Mashhad, Iran; 2 Department of Periodontology, School of Dentistry, Mashhad University of Medical Sciences, Mashhad, Iran; 3 Immunology Research Center, Inflammation and Inflammatory Diseases Division, Mashhad University of Medical Sciences , Mashhad, Iran; 4 Department of Periodontology, School of Dentistry,Gilan University of Medical Sciences, Rasht, Iran; 5 Departement of Periodontology, School of Dentistry, North Khorasan University of Medical Sciences, Bojnord, Iran; 6 Department of Periodontology, School of Dentistry, Babol University of Medical Sciences, Babol, Iran; 7 Department of Hygiene, Mashhad University of Medical Sciences, Mashhad, Iran

**Keywords:** Cytokine, Chronic periodontitis, Inflammatory mediators, Smoking, Interleukin, IL-12 gene, IL-1β

## Abstract

**Aim:**

The purpose of this study was to compare IL-1β and IL-12 gene expression in the gingival tissue of smokers and non-smokers either with healthy periodontium or with chronic periodontitis.

**Materials and Methods:**

41 individuals consisting of 21 healthy controls (11 non-smokers and 10 smokers) and 20 chronic periodontitis patients (10 non-smokers and 10 smokers) participated in this study. Samples were collected from papillary regions of targeted areas and cytokines were analyzed using Real Time PCR. Shapiro-Wilk, Mann-Witney and Independent T tests were employed for statistical analysis.

**Results:**

IL-1β gene expression in gingival tissue of non-smoker group with chronic periodontitis was significantly higher than non-smoker-healthy group (*p*=0.011). Smoker-chronic periodontitis group showed lower IL-1β gene expression than non-smoker-chronic periodontitis group (*p*=0.003). IL-12 gene expression was not significantly different between analyzed groups.

**Conclusion:**

IL-1β gene expression increases in gingival tissue of non-smoker-chronic periodontitis patients due to inflammatory processes but smoking reduces the expression of this cytokine in diseased periodontal tissues. On the other hand periodontal condition and smoking habits do not seem to affect IL-12 gene expressions in gingival tissues. Authors concluded that reduced levels of IL1 and in some extent IL12 in smoking patients are responsible for higher tissue and bone degenerations and less treatment responses in smokers.

## 1 INTRODUCTION

Smoking acts as a natural and valuable model for studying the pathogenesis of periodontal patients. The differences in periodontitis incidence and severity between smokers and non-smokers have been well documented [1]. In addition, it has been proved that periodontal response in smokers compared to non-smokers is unfavorable [2-4]. On the other hand, the mechanisms of these differences are not clearly identified. The associated studies show that the gingival inflammation in smokers is less than non-smokers. In the case of severe periodontitis, smoker’s gingiva shows inflammation symptoms such as redness and bleeding to less extent [5]. A major portion of connective tissue and bone destruction, as a result of periodontitis, is due to pro-inflammatory cytokines originating from the host periodontal tissues. Reduced inflammatory cytokine level in smokers compared to periodontitis patients should be answered. The question is that which one of the numerous immune-inflammatory mechanisms in periodontium plays a protective role and which one plays the destructive one. The comparison of existing cytokines in the gingiva of healthy individuals, smoking and non-smoking patients might potentially offer valuable information regarding the primary mechanisms of connective tissue destruction and alveolar bone loss in periodontitis.

IL1β plays a significant role in inflammation and immunity. IL-1βis mainly produced by monocytes, macrophages and neutrophils and also by other cell type such as fibroblasts and epithelial cells. IL1β increase expression of (Intra Cellular Adhesion Molecule) ICAM and secretion of chemokines. It also synergizes with other pro-inflammatory cytokines in bone resorption. It has a role in adaptive immunity, regulates antigen presenting cells and has shown to enhance antigens mediated of T-cells [6].

IL-12 has several immunological activities. Early and powerful production of IL-12 during first phase of infection may activate macrophages and stimulate the host’s cell-mediated immunity which results to the antigen-specific immune responses. In humans, the major effect of IL-12 is to stimulate (Interferon Gamma) IFN-γ production by (T-helper1) Th1 cells and regulate the transition from an early innate immune response to an adaptive immune response [7].

The effect of nicotine on osteoclast production in human periodontal ligament (PDL) cultured with or without T-helper cells was evaluated by Wu *et al*. In mono-culture systems, the nicotine stimulation leads to increased expression of IL-1β in the serum of human PDL cells cultured with T-helper cells. In addition, the expression of receptor activator of nuclear factor ligand (RANKL) in human PDL cells in a medium co-cultured with T-helper cells was higher while expression of osteoprotegrine (OPG) in mono and co-cultured systems showed insignificant differences [8].

Toker *et al.* examined the effects of smoking on the IL-1β levels, oxidants and anti-oxidants in gingival crevicular fluid of patients with chronic periodontitis before and after treatment. The basic levels of IL-1β were significantly higher in smokers compared with non-smokers but after periodontal treatment, the IL-1β levels significantly reduced in the both groups. In addition, no significant difference in the general levels of oxidants and anti-oxidants before and after periodontal treatment was reported. The results of their study showed that the periodontal treatment improved the clinical parameters in smoker and nonsmoker patients. Also the effectiveness of periodontal treatment on IL-1β levels in gingival crevicular fluid was confirmed while the treatment had reportedly no influence upon the general condition of oxidants and antioxidants [9].

In another study it was found that the individuals with periodontitis had increased levels of cytokine and chemokine while in smokers the levels of pro-inflammatory cytokines and T-cell and Natural Killer cell (NK-cell) specific regulators decreased. This reduction in smokers might be due to immunosuppressive effect and increased risk of periodontitis [10].

Ozcaka *et al*. suggested that periodontal inflammation in smokers with chronic periodontitis is associated with reduced OPG level of plasma and increased RANKL/OPG ratio, both potentially affect the destruction of alveolar bone in smoking patients [11].

The review of the literature shows that more studies concerning the periodontal status of smokers and non-smokers in regard to genetic expression of cytokines are required. IL-1β has a fundamental role in the pathogenesis of periodontitis and IL 12 has regulatory effect. Changing the level of these cytokine with the effect of smoking might alter the clinical feature and treatment response of periodontitis. Therefore, in the present study, the effects of smoking on expression of IL-12 and IL-1β in the gingival tissue of people with chronic periodontitis were evaluated.

## 2 MATERIALS AND METHODS

Among the patients referred to the Periodontology Department of Mashhad Dental School for periodontal and crown lengthening surgery, those who had inclusion criteria were recruited and examined after signing an informed consent. For smoking criterion, the patients should have smoked 20 cigarettes per day for the past 10 years. The criteria for periodontitis were attachment loss and minimum probing depth of 6mm or higher in at least eight sites, along with BOP and radiographic bone loss. The data were collected in a field-based manner and among 41 patients, 21 patients were healthy (11 non-smokers and 10 smokers) and the remaining 20 patients had chronic periodontitis (10 smokers and 10 non-smokers). Exclusion criteria were as follows: having any systemic diseases or any condition that might interact with periodontal disease, such as diabetes, HIV infection, Papillon-Lefèvre syndrome, Ehlers-Danlos syndrome, hypophosphatasia, any bacterial or viral infection and autoimmune diseases.

In patients with periodontitis, biopsy was obtained from buccal area of the deepest proximal pocket and through a horizontal cut with 5mm distance from palatal gingiva. The biopsies were removed from pockets with similar depth. In patients with normal gingiva and without periodontitis, similar biopsy of gingival tissue was obtained during crown lengthening surgery for prosthetic treatments. Meanwhile, it is endeavored to get biopsies similar in size. The weight of each biopsy was measured by a sensitive balance.

### 2.1 RNA Extraction and Real-Time PCR

Tissue samples were harvested and transferred to a tube containing RNA later solution (Qiagen, Germany) kept at –20°C so the mRNA levels of IL-12 and IL-1β could be measured. Total RNA was extracted from samples using an RN easy mini-kit (Qiagen, Germany) according to the manufacturer’s instructions. The RNA was then reverse transcribed to complementary DNA (cDNA) with oligo-dT primers on 1 µg of total RNA using First Strand cDNA Synthesis Kit (Fermantase, Germany). Real-time PCR was performed on the cDNA samples using Premix Ex taq (TAKARA, Japan) and Rotor-Gene 6000 system (Corbett, Australia). The sequences of primer pairs for real-time PCR were:

IL-1β: Forward

5’- CTG GAC CTC TGC CCT CTG

Reverse

5’- CAG CAT CTT CCT CAG CTT GTC

IL-12: Forward

5’- GCTTCT TCA TCAGGG ACA TC

Reverse

5’- CTC CAG GTG TCA GGG TAC TC

β2M: Forward

5’- TTG TCT TTC AGC AAG GAC TGG

Reverse

5’- CTC CCA CTT AAC TAT CTT GGG CTG TG

The amplification of a single product for each primer set was confirmed by electrophoresis analysis on a 2% agarose gel and melting curve in real-time PCR. Serial dilution standard curves were developed for target and reference genes to relatively quantify the copy number of each single gene. The Rotor-Gene 6000 machine and its software were used to analyze the standards and the unknown mRNA copy number. The relative quantity of each mRNA was normalized to the relative quantity of β2M mRNA. Then the relative IL expression levels for each sample were calculated by an equation of:

IL-1β or IL-12 Normalized Index = copy number of gene of interest (IL-1 or 12) / copy number of reference gene (β2M).

### 2.2 Statistical Analysis

The differences for IL-1β and IL-12 between three groups (control, smokers and non-smokers chronic periodontitis) were analyzed using Shapiro-Wilk, Mann-Whitney U test and independent T-tests. The differences were considered significant if *p* < 0.05.

## 3 RESULTS

As it is observed in Table **1**, the amount of IL-1β expression in nonsmokers was higher than smokers in both healthy and periodontitis groups as well as the whole population but the difference is significant only in the periodontitis group (*p*=0.003). In addition the amount of IL-1β in nonsmoker healthy group was statistically lower than nonsmoker periodontitis patients (*p*=0.011). When we compared this value between two groups, regardless of smoking, we found that the amount of IL-1β was significantly higher in periodontitis patients (*p*=0.027) than healthy groups.

IL-12 did not show any significant difference between smoker and nonsmokers in both healthy and periodontitis group (*p*=0.778 and *p*=0.940, respectively).

Table **2** shows that the amount of IL-12 in periodontitis group is less than the healthy in both groups of smokers and nonsmokers as well as the whole population, but the differences are not significant. Figs. (**1** and **2**) show the values of IL-1β and IL-12 in individuals with healthy periodontal tissues and in smoker and nonsmoker patients with periodontitis.

“Therefore, we can conclude that the reduction of IL-12 may lead toward periodontitis. Although in our study the reduction was not significant. Probably if we increase the sample size, we could find significant results.”

## 4 DISCUSSION

In this study, levels of IL1β and IL12 in normal and gingival tissues with periodontitis in smokers and nonsmokers were determined through Real-time PCR method.

Patients in the present study passed the first phase of therapy in periodontal treatment plan. Buccal cells are an excellent source of DNA for diagnosis and large-scale molecular epidemiological studies which are simple and cost effective methods in comparison to paper for (Gingival Crevicular Fluid) GCF collection. The differences between cytokines in biopsies and GCF may be due to the lack of cytokine detection in GCF because of the high specificity of the applied techniques. The absence of cytokine detection in saliva and GCF could be related to the fact that the cytokines secreted were bound to their membrane receptors and thus remained in the tissues; Cytokines that would be released to the GCF but would be consumed by regulatory processes; or would not be absorbed by the absorbent paper. Thus, it seems plausible that the gingival fluid not exactly express the markers [12].

The absence of cytokine detection in saliva and GCF can be due to several reasons; cytokines remained bounded to their membrane receptor after secretion thus remaining in the tissues or after the cytokine secretion into the GCF they would be consumed by regulatory processes causing them not to be absorbed by absorbent paper. It seems plausible that the gingival fluid not exactly expresses the markers [12].

The findings of this study show that IL-1β gene expression among those patients with periodontitis (excluding smoking cigarette) is significantly higher than normal individuals. This could be justified considering the pro-inflammatory essence of this cytokine and that of the periodontal disease. In this regard Teles (2010) reported increased values of pro-inflammatory cytokines such as IL-1β in GCF of patients with periodontitis [13]. This issue (increased values of IL-1β) is even observed in non-diseased sites with low probing depth in patients with severe periodontitis forms which led to considering patients and genotype factors that involved in host’s response to bacterial coping. Therefore, there is no doubt that this cytokine plays a determining role in emergence of inflammatory events during periodontitis.

This study suggests that smoking cigarettes in patients with periodontitis significantly reduces IL-1β gene expression in comparison to nonsmoking patients. This is aligned with lower intensity of inflammatory symptoms of gingiva in smoking patients [14]. However, it is presumed that despite seeing less inflammatory signs of periodontal diseases in smoking patients, the degree of periodontal destruction and bone loss in these patients are higher than nonsmoking patients.

The results of different studies concerning the effects of cigarette on IL-1β level in individuals with periodontitis are controversial. In agreement with this study Rawlinson and Torres de Heens reported reduced levels ofIL-1β in GCF of smoking individuals with periodontitis [15, 16]. Some studies such as Bostrom reported lack of change and others such as Toker reported increased levels of cytokines [9, 17]. As Rawlinson suggested, that reduced levels of IL-1β in GCF of smoking patients with periodontitis are probably related to tissue receptors.

The higher amount of periodontal destruction in smokers which was stated by Rawlinson, was not considered in this present study. In other words, the IL-1β levels are even lower in the tissues of smokers. This might show the lower cytokine production in periodontal diseases [15].

In addition, the IL-12 gene expression was also analyzed but there were no significant differences observed in our study. On the other hand the levels of IL-12 were also measured. The IL-12-IFNy pathway could enhance bone degeneration as a result of inflammatory processes and also inhibit it through direct influence on osteoclasts. Consequently, a dual action is presumed for this pathway [18]. However, some studies consider this cytokine as a member of pro-inflammatory cytokines for inflammatory diseases such as periodontitis [19]. The reported levels for this cytokine were similar to IL-1β but varied in different studies. The results of our study showed lower level of IL-12 in patients with periodontitis compared to normal individuals, but the difference between them was not significant.

Some researchers believe that reduced values of IL-12 paired with the increased values of IL-18 might shift the response of T-lymphocyte toward T-helper 2 phenotype and have reported this event in periodontitis [20]. There is increasing evidence that periodontal damages are directed by T-helper 1 lymphocytes. In contrast, progressive damages are due to T-helper 2 lymphocytes. In the presence of IL-12, IL-18 responses are modulated towards T-helper 1 whilst lack of these cytokines leads the response to T-helper 2 [21]. Therefore, we can conclude that the reduction of IL-12 may lead toward periodontitis. Although in our study the reduction was not significant. Probably if we increase the sample size we could find significant results. Increasing sample size could probably yield significant results.

Some studies reported significant reduction of IL-12 levels in the GCF of smoking patients with periodontitis compared to nonsmokers [10]. Others, such as this study, did not report significant difference in the expression of this gene [16]. However, reduced values of this cytokine in GCF seem to be due to the fact that the GCF expression level of smokers is less [14], but, this concentration reduction cannot solely represent the reduced production of this cytokine.

The measurement of interleukins level in GCF through ELISA method (a common method in majority of the above-mentioned studies) has limitations including: sampling sensitivity, GCF collection duration, inhibition of saliva contamination and other fluids such as blood, and difficulty in sampling and GCF evaporation [22]. We maintained the mRNA content of tissue for a relatively long duration through RNA Later solution at -20°C (normal freezer). In addition, mRNA content of tissue through this method can be directly measured (Instead of indirect measurement of cytokine content of GCF).

Another noteworthy point is that periodontitis samples in the present study belong to the patients that had passed the first phase of therapy in periodontal treatment plan; therefore, the tissue inflammation intensity was less than when the samples were prepared from a completely inflamed gingiva. This issue might have an influence on reporting less values of inflammatory cytokines.

## CONCLUSION

The level of IL-1β gene expression among nonsmoker periodontitis patients compared to nonsmoker healthy individuals was significantly higher. In addition, the level of IL-1β gene expression in smokers was significantly less than nonsmokers in periodontitis group. The level of IL-12 gene expression showed no significant differences in all of the studied groups.

Authors concluded that reduced levels of IL1 and in some extent IL12 by smoking patient are responsible for higher tissue and bone degeneration and less treatment response in smokers.

## Figures and Tables

**Fig. (1):**
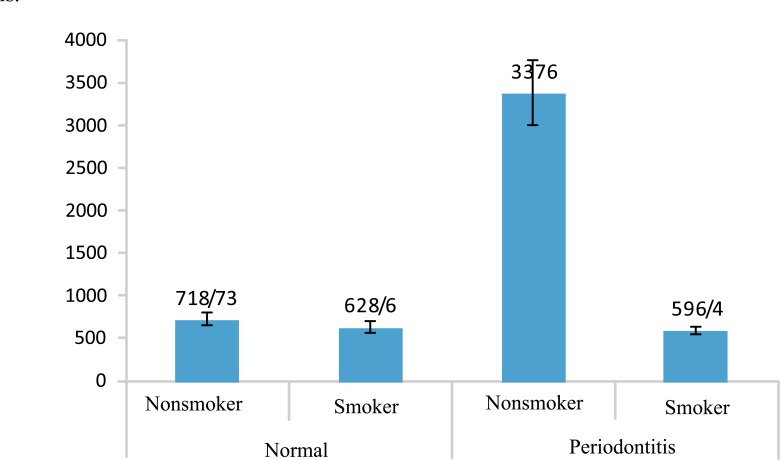
Mean IL-1β values of smokers and non-smokers based on periodontal status (normal and periodontitis).

**Fig. (2):**
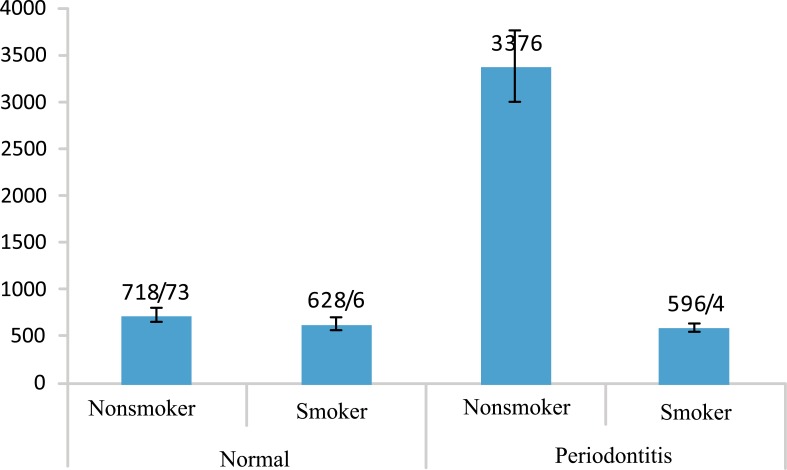
Mean IL-12values of smokers and non-smokers based on periodontal status (normal and periodontitis).

**Table 1:** Comparison of Mean IL-1β between Smokers and Nonsmokers based on Periodontal Status (Healthy and Periodontitis).

Group	Smoking	N	Mean	SD*	Test Result
Healthy	No	11	718.73	840.75	Z=-.211 *P*=0.833
	Yes	10	628.60	2627.32	
	Sum^	21	1675.81	3729.83	
Periodontitis	No	10	3376.00	3851.11	Z=-2.948 *P*=0.003
	Yes	10	596.40	416.12	
	Sum	20	1986.2	3023.32	
Total Population	No	21	1984.09	2979.39	Z=-1.747 *P*=0.081
	Yes	20	612.50	518.36	
	Sum	41	1315.02	2246.73	

*SD: Standard deviation

^ Sum: total

**Table 2:** Comparison of Mean IL-12 between Healthy and Periodontitis patients based on Smoking Status (Smoker and Nonsmoker).

Group	Periodontal Status	N	Mean	SD	Test Result
Nonsmoker	Healthy	11	246.09	372.62	Z=-0.775 *P*=0.438
	Periodontitis	10	123.70	160.13	
	Sum	21	187.81	291.35	
Smoker	Healthy	10	248.90	312.99	Z=-0.68 *P*=0.496
	Periodontitis	10	152.00	157.39	
	Sum	20	200.45	246.19	
Total Population	Healthy	21	247.43	336.91	Z=-1.043 *P*=0.297
	Periodontitis	20	137.85	155.21	
	Sum	41	193.97	266.97	

## LIST OF ABBREVIATIONS

IL1β: Interleukin 1 beta
ICAM: Intracellular adhesion molecule
GCF: Gingival crevicular fluid
INF: Interferon
TH1: T lymphocyte helper 1
NK cell: Natural killer Cell
OPG: Osteoprotegrin
RANK: Receptor Activator of Nucleic Factor κ B
PCR: polymerase chain reaction
